# Intervenção Coronária Percutânea Induziu No-reflow em Enxertos de Veia Safena

**DOI:** 10.36660/abc.20240317

**Published:** 2024-09-10

**Authors:** Yücel Kanal

**Affiliations:** 1 Cumhuriyet University Faculty of Medicine Sivas Turquia Cumhuriyet University Faculty of Medicine – Cardiology, Sivas – Turquia

**Keywords:** Fenômeno de não Refluxo, Veia Safena, Intervenção Coronária Percutânea

**Caro Editor,**

Li com interesse o estudo intitulado "O valor preditivo do índice prognóstico inflamatório para detecção de no-reflow em pacientes com infarto do miocárdio com supradesnivelamento do segmento ST",^1^ publicado em sua revista. Gostaria de contribuir com o estudo fornecendo alguns insights.

O estudo avaliou a relação entre vários parâmetros inflamatórios e no-reflow em pacientes submetidos a intervenção coronária percutânea (ICP) para infarto do miocárdio com supradesnivelamento do segmento ST.^1^ O fenômeno de no-reflow observado durante a ICP manifesta-se como uma redução ou cessação abrupta do fluxo sanguíneo coronariano anterógrado, apesar da ausência de fatores precipitantes claros, como espasmo, dissecção, macrotrombo distal, trombose in situ ou estenose coronariana residual.^2^ Apesar de alcançar precisão técnica na ICP, ainda podem ocorrer casos de comprometimento do fluxo após a colocação do stent. Especialmente em pacientes com enxertos de veia safena (EVS) degenerados, há uma tendência aumentada para o desenvolvimento do fenômeno de no-reflow.^3^ Estudos indicaram incidências variadas de no-reflow durante ICP, variando entre 12% e 25%, com exame específico de EVS em ICP revelando taxas entre 15% e 42%.^4^ Neste estudo, não há dados fornecidos quanto à inclusão de pacientes submetidos à ICP de EVS. Além disso, o estudo não contém informações sobre a relação entre ICP EVS e no-reflow. Portanto, contribuirei com alguns *insights* sobre o desenvolvimento do no-reflow seguindo ICP EVS.

Em nosso estudo que investigou a relação entre a relação proteína C reativa/albumina (CAR) e o no-reflow em pacientes submetidos à ICP EVS, observamos taxas significativamente mais altas de no-reflow em pacientes com níveis elevados de CAR. Nossa taxa geral de no-reflow foi determinada como 19,8%, enquanto no subgrupo com síndrome coronariana aguda (SCA) foi de 25,6%.^4^ Essas taxas são inferiores às relatadas em estudos anteriores pelos autores (11,5%).^1^ Acredito que a provável razão para isso seja a ausência de pacientes submetidos à ICP-EVS no estudo. Em estudo que avaliou a relação entre o índice de imunoinflamação sistêmica (SII) e a taxa de no-reflow em pacientes com SCA submetidos à ICP-EVS, observou-se que pacientes com índices de SII mais elevados apresentaram taxas significativamente maiores de no-reflow.^5^ Em outro estudo que realizamos anteriormente, se observou que os pacientes com escores de trombo TIMI (Thrombolysis in Myocardial Infarction) mais altos no vaso antes da ICP de EVS tinham taxas significativamente mais altas de no-reflow entre os que apresentavam SCA.^6^

Concluindo, existe um alto risco de desenvolvimento de no-reflow na ICP EVS. Portanto, especificar a proporção de pacientes com ICP EVS entre os grupos em estudos que avaliam o no-reflow aumentará a força do estudo.
