# Supplementary material for: Intervenção Coronária Percutânea Induziu No-reflow em Enxertos de Veia Safena
Source: Arq Bras Cardiol. 2024 Sep 10;121(8):e20240317. [Article in Portuguese] doi: 10.36660/abc.20240317 (PMC12092045; doi:10.36660/abc.20240317)
Supplement: Translation

# Translation: Percutaneous Coronary Intervention Induced-No-reflow in Saphenous Vein Grafts

**DOI:** 10.36660/abc.20240317i

**Authors:** Yücel Kanal

**Affiliations:** 1 Sivas Turkey Cumhuriyet University Faculty of Medicine – Cardiology, Sivas – Turkey

**Keywords:** No-Reflow Phenomenon, Saphenous Vein, Percutaneous Coronary Intervention

**Dear Editor,**

I have read with interest the study titled "The Predictive Value of the Inflammatory Prognostic Index for Detecting No-Reflow in ST-Elevation Myocardial Infarction Patients",^1^published in your journal. I would like to contribute to the study by providing some insights.

The study evaluated the relationship between various inflammatory parameters and no-reflow in patients undergoing percutaneous coronary intervention (PCI) for ST-elevation myocardial infarction.^1^The no-reflow phenomenon observed during PCI manifests as a reduction or abrupt cessation of antegrade coronary blood flow, despite the absence of clear precipitating factors such as spasm, dissection, distal macrothrombus, in situ thrombosis, or residual coronary stenosis.^2^Despite achieving technical precision in PCI, instances of flow impairment may still ensue following stent placement. Especially in patients with degenerated saphenous vein grafts (SVG), there is an increased tendency for the development of the no-reflow phenomenon.^3^Studies have indicated varying incidences of no-reflow during PCI, ranging between 12% and 25%, with specific examination of SVG in PCI revealing rates between 15% and 42%.^4^In this study, there is no provided data regarding the inclusion of patients undergoing SVG PCI. Furthermore, the study does not contain information regarding the relationship between SVG PCI and no-reflow. Therefore, I will contribute some insights regarding the development of no-reflow following SVG PCI.

In our study investigating the relationship between the C-reactive protein to albumin ratio (CAR) and no-reflow in patients undergoing SVG PCI, we observed significantly higher rates of no-reflow in patients with elevated CAR levels. Our overall no-reflow rate was determined as 19.8%, while in the subgroup with acute coronary syndrome (ACS), it was 25.6%.^4^These rates are lower than those reported in previous studies by the authors (11.5%).^1^I believe the probable reason for this is the absence of patients undergoing SVG-PCI in the study. In a study evaluating the relationship between the systemic immune-inflammation index (SII) and the rate of no-reflow in patients with ACS undergoing SVG PCI, it was observed that patients with higher SII indices had significantly higher rates of no-reflow.^5^In another study we conducted previously, it was observed that patients with higher Thrombolysis in Myocardial Infarction (TIMI) thrombus scores in the vessel before SVG PCI had significantly higher rates of no-reflow among those presenting with ACS.^6^

In conclusion, there is a high risk of developing no-reflow in SVG PCI. Therefore, specifying the proportion of SVG PCI patients among groups in studies evaluating no-reflow will enhance the strength of the study.
